# Human resource allocation status and equity research of Centers for Disease Control and Prevention in China from 2016 to 2020

**DOI:** 10.3389/fpubh.2024.1382343

**Published:** 2024-08-29

**Authors:** Shen Shao, Kunzai Niu, Xinye Qi, Fangshi Li, Li Wang, Yawei Sheng, Xinyu Qiu, Yi Li, Yue Du, Haojun Fan

**Affiliations:** ^1^Institute of Disaster and Emergency Medicine, Tianjin University, Tianjin, China; ^2^College of Management and Economics, Tianjin University, Tianjin, China; ^3^School of Public Health, Tianjin Medical University, Tianjin, China

**Keywords:** Centers for Disease Control and Prevention, Gini coefficient, Theil index, health resource density index, allocation of human resources, China

## Abstract

**Background:**

In recent years, the development of global public health has become a matter of great concern and importance for governments worldwide. China, as the largest developing country, plays a crucial role in shaping the development of the public health and its ability to respond to sudden public health emergencies through the fairness of its human resource allocation in center for disease control and prevention (CDC).

**Objective:**

This study aims to analyze the situation of health human resource allocation in the China Centers for Disease Control and Prevention (China CDCs), assess the fairness of the allocation, and provide reference for the rational allocation of human resources.

**Methods:**

We selected data from the China Health Statistics Yearbook on healthcare technical personnel, other technical personnel, managerial personnel, and workforce technical personnel of China CDCs for the period of 2016–2020. We utilized the Health Resource Density Index to evaluate the level of human resource allocation in China CDCs. Additionally, we used the Gini coefficient and Theil index to assess the fairness of human resource allocation in China CDCs from both a population and geographical perspective.

**Results:**

Firstly, the educational qualifications and professional titles of CDC staff have improved, but the workforce is aging. Secondly, HRDI development trends vary among different personnel types and regions with varying levels of economic development. Finally, the results of the Gini coefficient and Theil index indicate that population distribution fairness is better than geographical distribution fairness. Overall, the unfair population distribution is primarily due to regional disparities.

**Conclusion:**

The China CDCs should tailor different standards for the allocation of health human resources based on regional characteristics, aiming to enhance the accessibility of health human resources in various regions and achieve equitable allocation.

## Introduction

**Figure 1:**
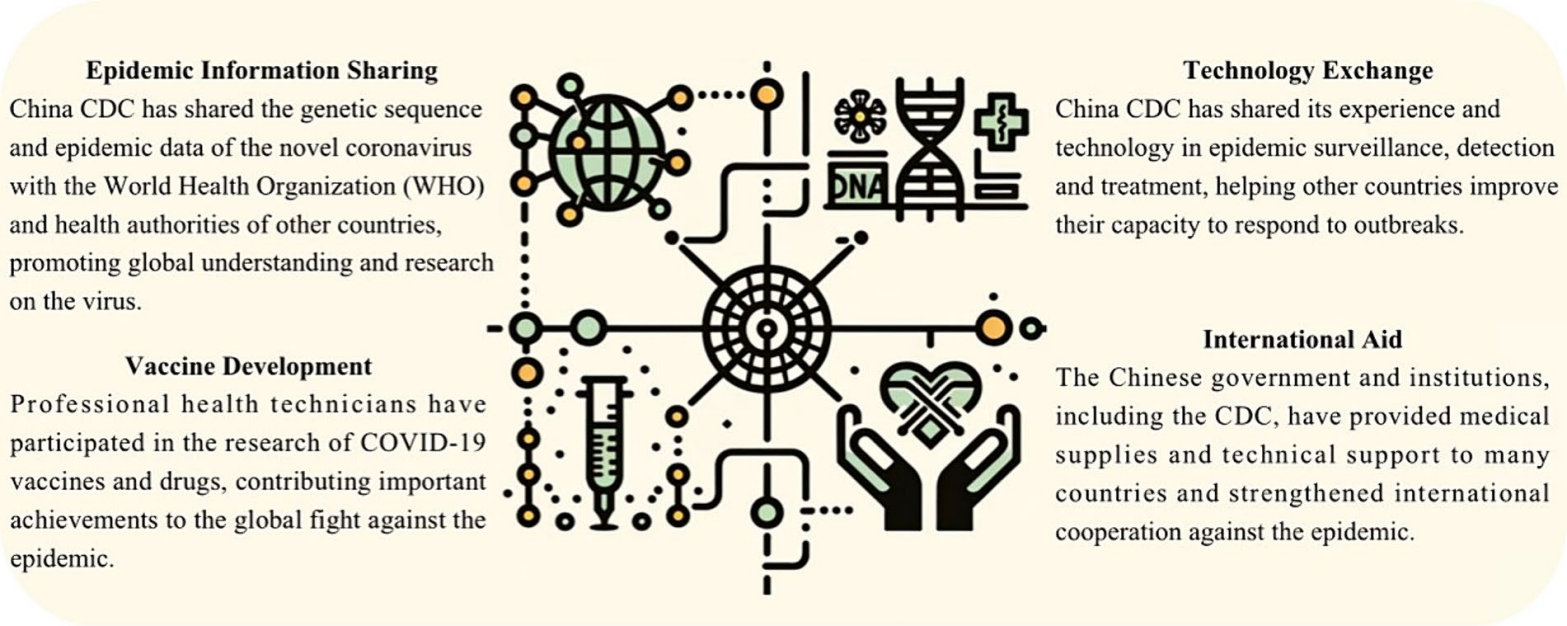
The work of the China CDC for the international community’s fight against COVID-19.

In recent years, the development of public health has become a field of great concern and attention by governments around the world (1). As one of the core agencies in the field of public health, center for disease control and prevention (CDC) undertakes important responsibilities such as disease prevention, control of infectious sources, and provision of public health services (2–4). At the current stage, the normalization of global epidemic prevention and control has intensified the demand for public health talent (5). However, the construction of the disease control system is a long and arduous task, especially with new requirements and tasks emerging. Figure 1 shows the various contributions made by the China CDC to the international community in the fight against the coronavirus disease 2019 (COVID-19) pandemic. Through various channels and international cooperation, China CDC has worked closely with the World Health Organization (WHO) to address this global public health crisis (6, 7).

The work of the CDC requires talents from multiple professional fields (8, 9), and the fairness of its human resources is closely related to the equity of public health service utilization (10, 11). Balanced allocation of CDC human resources is beneficial for ensuring the equitable implementation of public health services (12) and plays a crucial role in building a public health service system that is adapted to the level of national economic and social development and matches the health needs of the people (13). However, there is limited research on the changes in the composition of CDC human resources and the fairness of allocation of various types of health human resources in China. As the world’s largest developing country, China has accumulated rich experience in addressing public health challenges (14, 15). By studying the human resource allocation of China’s CDCs, effective management strategies and best practices can be identified to enhance the ability to respond to sudden public health events. This is of great significance for improving the global public health system, promoting international health cooperation, and supporting sustainable development goals.

This study comprehensively evaluated the fairness of the allocation of human resources in China CDCs during the “13th Five-Year Plan” period (2016–2020) based on both population and geographical area, utilizing the Gini coefficient (G), Health Resource Density Index (*HRDI*), and Theil index (T).

The *HRDI* was used to reflect the comprehensive level of CDC personnel allocation, taking into account both population and geographical factors. The G and T were employed to assess the fairness of CDC staff distribution based on population and geographical area, respectively. This approach allowed for a multi-dimensional evaluation of the fairness of personnel allocation in China CDCs across different regions.

The findings of this study aim to provide decision-making references for the optimization of human resource allocation in China CDCs during the “14th Five-Year Plan” period, contributing to the improvement of China’s public health standards and the well-being of global health.

## Data collection and research methods

### Data sources

Data on the healthcare human resources at the China CDCs (including healthcare technical personnel, other technical personnel, managerial personnel, and workforce technical personnel, etc.) were sourced from the “China Health Statistical Yearbook.” The research indicators included the number of CDC personnel and their qualifications’ structure from 2016 to 2020, such as age, educational level, years of work experience, and professional titles.

Geographical area and population distribution data were obtained from the “Administrative Division Handbook of the People’s Republic of China” and the “China Statistical Yearbook.” The study covered administrative regions in mainland China, encompassing 31 provincial-level administrative divisions.

### Functional regional division

Based on the *per capita* Gross Domestic Product (GDP) levels, mainland China is divided into three regions (16):

1. Eastern Region, comprising 11 provinces: Beijing, Tianjin, Hebei, Liaoning, Shanghai, Jiangsu, Zhejiang, Fujian, Shandong, Guangdong, and Hainan.
2. Central Region, comprising 8 provinces: Shanxi, Jilin, Heilongjiang, Anhui, Jiangxi, Henan, Hubei, and Hunan.
3. Western Region, comprising 12 provinces: Chongqing, Sichuan, Guizhou, Yunnan, Tibet, Shaanxi, Gansu, Qinghai, Ningxia, Xinjiang, Guangxi, and Inner Mongolia.

### Measurements of allocation status and equity

The G is widely used to evaluate the overall fairness of healthcare resource allocation but cannot provide regional breakdowns of inequality (17). The T can reflect differences in fairness within and between regions, allowing for the analysis of whether the source of inequality is primarily due to differences within regions or between regions (18). The *HRDI* is applied in the assessment of healthcare resource allocation fairness, considering both population and geographical factors. It provides a better reflection of the comprehensive levels of healthcare resource distribution based on both population and geographical area (19).

Based on the above analysis, this study comprehensively utilizes analytical methods such as the *HRDI*, the *G* and the *T* to analyze the fairness of healthcare resources in the each region in China from both a population and geographical perspective. The *HRDI* is used to measure the level of CDC human resource allocation in different economic regions. Fairness of distribution is evaluated based on the calculated G and T. The research results reflect the development of China CDCs human resources during the “Thirteenth Five-Year Plan” period.

#### Health resource density index

The health resource density index (*HRDI*)*, proposed by Zheng and Ling,* is a comprehensive indicator that assesses the level of healthcare resource allocation across both population and geographic regions. It can be employed to evaluate the distribution of healthcare resources in China (20). *HRDI* takes into account the influence of both population and geographic factors on the density and equity of medical resources, thus circumventing the limitations associated with solely considering population factors (21, 22). The calculation formula is as follows:


$$\mathrm{HRDI}=\sqrt{\frac{HR_{i}}{A_{i}}}\times \sqrt{\frac{HR_{i}}{P_{i}}}$$

*HR_i_*: The amount of healthcare resources in the ith province, including health technicians, other technicians, management personnel, and technical personnel (unit: person).
*A_i_*: The geographical area of the ith province (unit: square kilometers).
*P_i_*: The resident population in the ith province (unit: 1,000 people).
*HRDI*: The numerical value of the Health Resource Density Index.

Since the 1990s, there have been numerous studies in China that have focused on evaluating the status of medical resources and demonstrating their fairness within regional contexts (23, 24). This study introduces population quantity and land area as crucial factors in equity assessment, especially in countries with large populations and extensive land areas like China, where both population and geographic factors can significantly influence the fairness of healthcare resource allocation. A higher *HRDI* indicates a higher level of CDC human resource allocation in that region.

#### Gini coefficient

**Figure 2:**
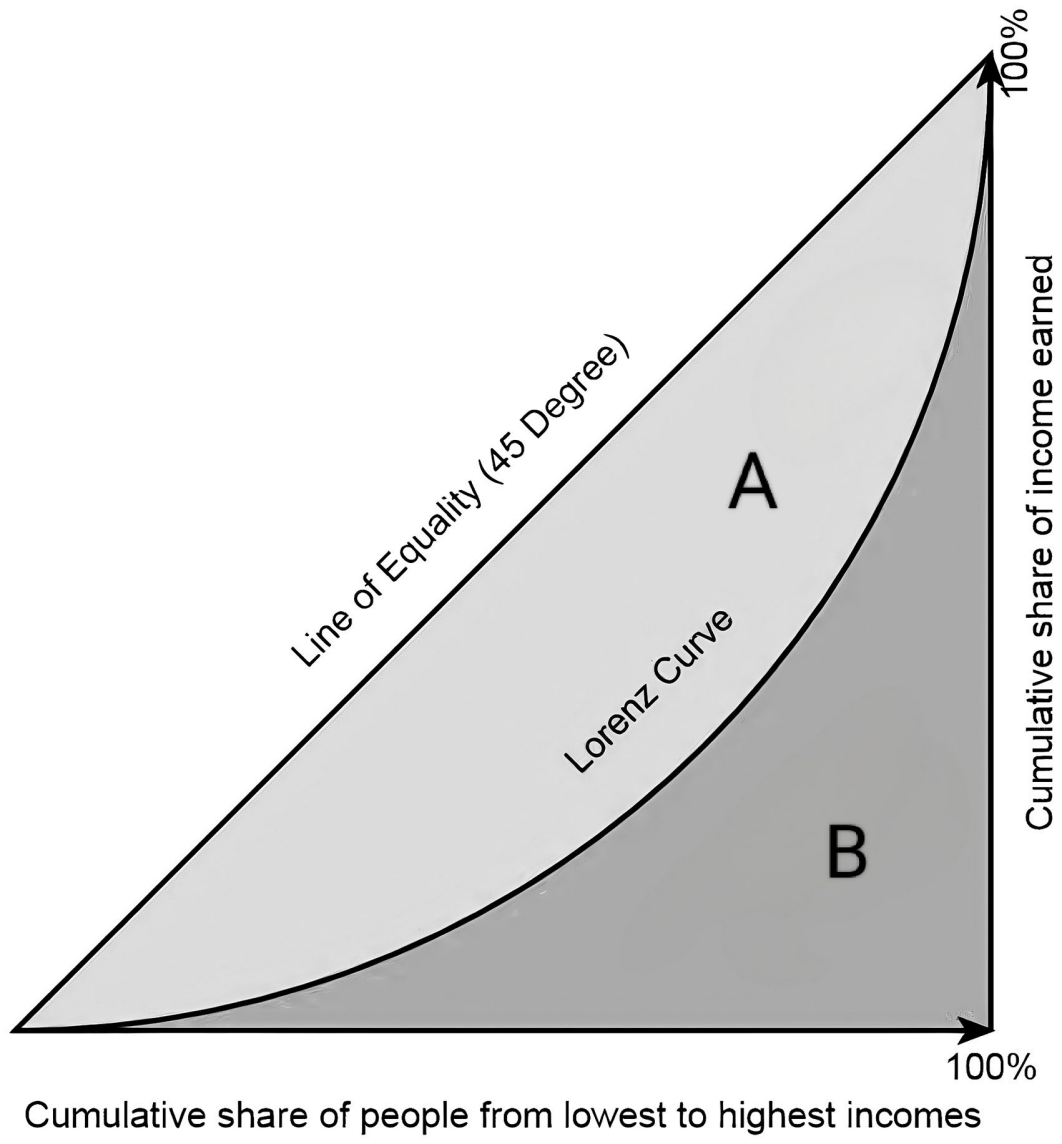
Lorenz Curve Illustration.

The G is a statistical indicator calculated based on the Lorenz curve, reflecting the degree of fairness in the distribution of social income (25). Currently, the G has been widely applied in assessing the fairness of healthcare resource allocation. It is equal to the ratio of the area enclosed by the absolute equality line and the Lorenz curve to the area under the absolute equality line in the form of a right-angled triangle (Figure 2).

Unlike the Lorenz curve, which is only visual, the G allows for quantitative evaluation (26). It represents a numerical expression of the Lorenz curve, and its calculation formula is as follows:


$$G=1-\overset{n-1}{\underset{i=1}{\sum }}\left(X_{i+1}-X_{i}\right)\left(Y_{i+1}+Y_{i}\right)$$

*n*: The total number of regions.
*Y_i_*: The cumulative proportion of CDC human resources in the *ith* region.
*X_i_*: The cumulative proportion of the population (geographical area) in the *ith* region.

The G has a range from 0 to 1. If G approaches 0, it indicates a more equitable distribution of CDC human resources across regions. Conversely, if G approaches 1, it suggests a more concentrated distribution of CDC human resources, indicating a less equitable resource allocation. Generally, 0.4 is often considered a “warning line” for healthcare resource allocation gaps. A Gini coefficient below 0.3 is considered the best average state, between 0.3 and 0.4 is considered normal, above 0.4 is a warning sign, and exceeding 0.6 is considered a highly unequal and dangerous state (17, 27).

#### Theil index

The T proposed by the Dutch economist Theil in 1976, originally studied income inequality from the perspectives of information and entropy. It can also be used to measure the fairness of regional healthcare resource allocation (28). The T decomposes the overall disparity into between-group (T_inter_) and within-group (T_intra_) disparities, allowing it to identify fairness at different levels and within different groups. It is highly sensitive to resource allocation efficiency and possesses a decomposable nature, making it an ideal analytical tool for equity studies (29). The T is widely used for evaluating the fairness of healthcare resource allocation (30). Its calculation formula is as follows:


$$T=\overset{n}{\underset{i=1}{\sum }}\xi_{i}\log\frac{\xi_{i}}{\eta_{i}}$$

n: The total number of regions.
ξ_i_: The proportion of the population (geographical area) in the ith region to the total national population (geographical area).
η_i_: The proportion of CDC human resources in the ith region to the total national CDC human resources.

The T has a range from 0 to 1, where a larger T indicates worse fairness, and a smaller T indicates better fairness (31). The decomposition formula for T is as follows:


$$\begin{array}{l} T=T_{\mathrm{intra}}+T_{\mathrm{inter}}\\ T_{\mathrm{intra}}=\overset{k}{\underset{j=1}{\sum }}\xi_{j}T_{j} \end{array}$$

$$T_{\mathrm{inter}}=\overset{k}{\underset{j=1}{\sum }}\xi_{j}\ln\frac{\xi_{j}}{\eta_{j}}$$

$$\omega_{\mathrm{intra}}=T_{\mathrm{intra}}/T$$

$$\omega_{\mathrm{inter}}=T_{\mathrm{inter}}/T$$

T: Total disparity.
T_intra_: Within-group T, reflecting the allocation disparity within different groups.
T_inter_: Between-group T, reflecting the allocation disparity between groups.
k: Three different economic level regions.
ξ_j_: The proportion of the population (geographical area) in the jth economic level region to the national population (geographical area).
T_j_: The Theil index of the jth economic level region.
η_j_: The proportion of CDC human resources in the jth economic level region to the national CDC human resources.
ω_intra_: Within-group disparity contribution rate.
ω_inter_: Between-group disparity contribution rate.

The disparity in resource allocation between groups is attributed to differences in economic levels, while within-group disparities primarily reflect the influence of non-economic factors. Grouping by economic level in research helps better identify the impact of economic differences on resource allocation.

## Results

### Basic situation of personnel in CDC

#### Number and composition of personnel

Table 1 presents the overall allocation of CDCs human resources in mainland China from 2016 to 2020. The personnel trends in the eastern region are similar to the overall trend. In the central region, the quantity of healthcare human resources remained relatively stable with minor fluctuations in growth rates, despite a slight decrease, and overall growth was limited. In the western region, the number of healthcare human resources experienced significant fluctuations between 2016 and 2017 but showed growth thereafter, especially in 2020, with a Chain growth rate of 3.39%.

**Table 1:** The distribution of healthcare human resources in China CDCs from 2016 to 2020.

Year	National		Eastern region		Central region		Western region	
	Personnel number	Chain growth rate	Personnel number	Chain growth rate	Personnel number	Chain growth rate	Personnel number	Chain growth rate
	*N*/10^3^	%	*N*/10^3^	%	*N*/10^3^	%	*N*/10^3^	%
2016	191.627	0.37	67.639	0.09	61.425	−0.34	62.536	1.47
2017	190.730	−0.47	66.180	−2.16	60.931	−0.80	63.619	1.73
2018	187.826	−1.52	64.790	−2.10	59.797	−1.86	63.239	−0.60
2019	187.564	−0.14	64.324	−0.72	59.224	−0.96	64.016	1.23
2020	194.425	3.66	68.329	6.23	59.908	1.15	66.188	3.39

#### Quality structure of personnel

Table 2 reflects the structural changes in the quality of human resources in China CDCs across different dimensions, including age, years of work experience, educational qualifications, and professional technical qualifications. From 2016 to 2020, there was a noticeable increase in the proportion of healthcare technical personnel and management personnel aged 55–59 and 60 years and above. There was a shift toward higher educational qualifications among healthcare personnel, with an increase in the proportion of undergraduates and postgraduates. In terms of the job title, the proportion of healthcare technical personnel with the titles of “associate professor of treatment” and “professor of treatment” increased.

**Table 2:** Quality structure of health human resources in China CDCs from 2016 to 2020.

Category	Healthcare technical personnel/%		Other technical personnel/%		Managerial personnel/%	
	2016	2020	2016	2020	2016	2020
**Age**						
<25	1.6	1.7	2.0	1.4	0.8	1.0
25~34	22.8	20.6	28.6	22.9	18.3	14.5
35~44	31.8	28.9	32.4	32.6	28.3	27.7
45~54	33.8	31.8	29.1	30.4	39.2	34.8
55~59	6.9	12.8	5.8	9.5	9.7	15.9
≥60	3.1	4.3	2.1	3.2	3.6	6.0
**Years of work experience**						
<5	10.0	9.1	11.5	8.5	5.5	5.3
5~9	11.7	12.1	14.4	13	9.7	7.6
10~19	21.8	21.3	24.7	25.2	19.6	20.5
20~29	31.6	28.2	28.2	27.3	32.3	28.4
≥30	24.9	29.2	21.3	25.9	32.9	38.3
**Education**						
Postgraduate	5.7	7.1	4.8	5.8	3.2	4.2
Undergraduate	33.1	42.5	31.4	41.1	36.4	42.7
Junior college	36.3	32.9	39.4	35.4	40.9	37.3
Technical secondary school	22.1	15.9	16.5	12.6	13.3	10.8
High school and below	2.9	1.6	7.9	5.1	6.2	4.9
**Job title**						
Professor of treatment	2.5	3.4	0.7	0.7	2.3	2.2
Associate professor of treatment	8.6	11.0	4.5	5.7	7.1	5.7
Doctor in-charge	31.1	29.2	20.4	22.5	17.8	13.7
Doctor practitioner	31.6	30.9	26.3	25.8	14.3	12.8
Assistant doctor	15.3	15.8	24.8	24.3	10.5	11.2
Unknown	10.9	9.7	23.3	20.9	48.0	54.3

### Level of health human resources allocation in CDC

#### Health resource density index calculation results

Table 3 represents the *HRDI* for various categories in mainland China CDCs for the year 2020. Overall, Shanghai had the highest *HRDI* for health technical personnel and other technical personnel. Tianjin and Henan had the highest *HRDI* for managerial personnel and workforce technical personnel, respectively. The regions with the lowest *HRDI* for all four categories of personnel were all in the Tibet. Specifically, in the economically developed eastern regions, Shanghai had the highest *HRDI* for health technical personnel, other technical personnel and workforce technical personnel. Tianjin had the highest *HRDI* for managerial personnel. Hebei had the lowest *HRDI* for health technical personnel, and Guangdong had the lowest *HRDI* for other technical personnel. Zhejiang had the lowest *HRDI* for managerial and workforce technical personnel. In the central regions, Henan had the highest *HRDI* for all four categories of personnel. The lowest *HRDI* for health technical personnel and workforce technical personnel was in Heilongjiang, while Jiangxi had the lowest *HRDI* for other technical personnel, and Anhui had the lowest *HRDI* for managerial personnel. In the less economically developed western regions, Shaanxi had the highest *HRDI* for health technical and managerial personnel, while Sichuan had the highest *HRDI* for other technical personnel, and Guangxi had the highest *HRDI* for workforce technical personnel.

**Table 3:** *HRDI* of China CDCs in 2020.

	Healthcare technical personnel				Other technical personnel				Managerial personnel				Workforce technical personnel			
Category	N/10^3^ population	*N*/10^3^M^2^	HRDI	Rank	*N*/10^3^ population	*N*/10^3^M^2^	HRDI	Rank	*N*/10^3^ population	*N*/10^3^M^2^	HRDI	Rank	*N*/10^3^ population	*N*/10^3^M^2^	HRDI	Rank
**Eastern Region**																
Shanghai	0.0901	0.3557	0.1790	1	0.0182	0.0717	0.0361	1	0.0054	0.0214	0.0108	4	0.0054	0.0214	0.0108	2
Beijing	0.1412	0.1840	0.1612	2	0.0112	0.0146	0.0128	5	0.0101	0.0132	0.0116	2	0.0057	0.0074	0.0065	14
Tianjin	0.1157	0.1419	0.1281	3	0.0149	0.0183	0.0165	2	0.0154	0.0188	0.0170	1	0.0074	0.0091	0.0082	6
Jiangsu	0.0927	0.0765	0.0842	4	0.0142	0.0118	0.0129	4	0.0053	0.0044	0.0048	15	0.0080	0.0066	0.0072	9
Shandong	0.0871	0.0575	0.0708	6	0.0111	0.0073	0.0090	7	0.0085	0.0056	0.0069	10	0.0065	0.0043	0.0052	18
Hainan	0.1201	0.0356	0.0654	7	0.0116	0.0034	0.0063	11	0.0122	0.0036	0.0066	11	0.0182	0.0054	0.0099	4
Zhejiang	0.0738	0.0467	0.0588	9	0.0090	0.0057	0.0071	8	0.0038	0.0024	0.0030	25	0.0053	0.0034	0.0043	23
Fujian	0.0970	0.0332	0.0568	14	0.0116	0.0040	0.0068	10	0.0061	0.0021	0.0036	21	0.0136	0.0046	0.0079	8
Guangdong	0.0644	0.0451	0.0539	15	0.0074	0.0052	0.0062	14	0.0050	0.0035	0.0041	19	0.0095	0.0066	0.0079	7
Liaoning	0.0931	0.0272	0.0503	18	0.0115	0.0034	0.0062	13	0.0177	0.0052	0.0096	5	0.0103	0.0030	0.0055	17
Hebei	0.0764	0.0304	0.0481	21	0.0143	0.0057	0.0090	6	0.0076	0.0030	0.0048	14	0.0151	0.0060	0.0095	5
**Central Region**																
Henan	0.0958	0.0570	0.0739	5	0.0200	0.0119	0.0155	3	0.0146	0.0087	0.0113	3	0.0366	0.0218	0.0282	1
Hubei	0.1093	0.0339	0.0609	8	0.0124	0.0039	0.0069	9	0.0073	0.0023	0.0041	20	0.0094	0.0029	0.0052	19
Hunan	0.1049	0.0329	0.0587	10	0.0112	0.0035	0.0063	12	0.0096	0.0030	0.0054	13	0.0177	0.0055	0.0099	3
Jiangxi	0.0988	0.0267	0.0514	17	0.0064	0.0017	0.0033	23	0.0063	0.0017	0.0033	23	0.0109	0.0029	0.0057	16
Jilin	0.1362	0.0175	0.0488	20	0.0169	0.0022	0.0061	15	0.0216	0.0028	0.0077	7	0.0120	0.0015	0.0043	22
Shanxi	0.0968	0.0216	0.0457	22	0.0104	0.0023	0.0049	20	0.0160	0.0036	0.0075	8	0.0150	0.0033	0.0071	10
Anhui	0.0676	0.0295	0.0447	23	0.0062	0.0027	0.0041	22	0.0048	0.0021	0.0031	24	0.0055	0.0024	0.0036	25
Heilongjiang	0.1334	0.0093	0.0353	26	0.0189	0.0013	0.0050	18	0.0175	0.0012	0.0046	17	0.0127	0.0009	0.0034	27
**Western Region**																
Shaanxi	0.1328	0.0255	0.0582	11	0.0055	0.0011	0.0024	27	0.0191	0.0037	0.0084	6	0.0153	0.0029	0.0067	12
Yunnan	0.1640	0.0202	0.0576	12	0.0133	0.0016	0.0047	21	0.0064	0.0008	0.0023	27	0.0173	0.0021	0.0061	15
Guangxi	0.1247	0.0265	0.0575	13	0.0115	0.0024	0.0053	17	0.0072	0.0015	0.0033	22	0.0149	0.0032	0.0069	11
Guizhou	0.1129	0.0247	0.0529	16	0.0059	0.0013	0.0028	25	0.0148	0.0032	0.0069	9	0.0073	0.0016	0.0034	26
Sichuan	0.1195	0.0208	0.0498	19	0.0140	0.0024	0.0058	16	0.0115	0.0020	0.0048	16	0.0158	0.0027	0.0066	13
Ningxia	0.1306	0.0142	0.0430	24	0.0089	0.0010	0.0029	24	0.0058	0.0006	0.0019	29	0.0111	0.0012	0.0037	24
Chongqing	0.0683	0.0266	0.0426	25	0.0079	0.0031	0.0049	19	0.0090	0.0035	0.0056	12	0.0076	0.0030	0.0048	20
Gansu	0.1321	0.0073	0.0310	27	0.0098	0.0005	0.0023	28	0.0182	0.0010	0.0043	18	0.0186	0.0010	0.0044	21
Inner Mongolia	0.1897	0.0039	0.0270	28	0.0190	0.0004	0.0027	26	0.0169	0.0003	0.0024	26	0.0155	0.0003	0.0022	28
Xinjiang	0.1769	0.0028	0.0221	29	0.0144	0.0002	0.0018	30	0.0171	0.0003	0.0021	28	0.0162	0.0003	0.0020	29
Qinghai	0.2073	0.0017	0.0188	30	0.0204	0.0002	0.0018	29	0.0108	0.0001	0.0010	30	0.0199	0.0002	0.0018	30
Tibet	0.3045	0.0009	0.0166	31	0.0189	0.0001	0.0010	31	0.0178	0.0001	0.0010	31	0.0238	0.0001	0.0013	31

Table 4 shows the *HRDI* of CDCs for different economic regions in mainland China from 2016 to 2020. The *HRDI* for health technical personnel and other technical personnel exhibits an upward fluctuating trend, while the *HRDI* for managerial personnel and workforce technical personnel remains relatively stable with a smaller range of fluctuations. Across all categories of personnel, the eastern regions consistently maintain a higher *HRDI* compared to the western regions, where the *HRDI* is the lowest.

**Table 4:** *HRDI* of CDCs in different economic levels in mainland China from 2016 to 2020.

Category	2016	2017	2018	2019	2020
**Healthcare technical personnel**					
National	0.0391	0.0389	0.0383	0.0385	0.0395
Eastern Region	0.0652	0.0640	0.0625	0.0614	0.0641
Central Region	0.0506	0.0502	0.0493	0.0505	0.0505
Western Region	0.0303	0.0306	0.0306	0.0307	0.0317
**Other technical personnel**					
National	0.0040	0.0040	0.0041	0.0042	0.0046
Eastern Region	0.0076	0.0074	0.0074	0.0079	0.0086
Central Region	0.0064	0.0061	0.0063	0.0062	0.0066
Western Region	0.0021	0.0023	0.0023	0.0025	0.0027
**Managerial personnel**					
National	0.0038	0.0038	0.0037	0.0037	0.0038
Eastern Region	0.0059	0.0055	0.0054	0.0055	0.0055
Central Region	0.0057	0.0057	0.0056	0.0056	0.0056
Western Region	0.0028	0.0029	0.0028	0.0028	0.0029
**Workforce technical personnel**					
National	0.0005	0.0005	0.0004	0.0004	0.0004
Eastern Region	0.0003	0.0003	0.0003	0.0003	0.0003
Central Region	0.0006	0.0006	0.0006	0.0005	0.0006
Western Region	0.0005	0.0005	0.0005	0.0005	0.0005

#### Gini coefficient measurement results

**Figure 3:**
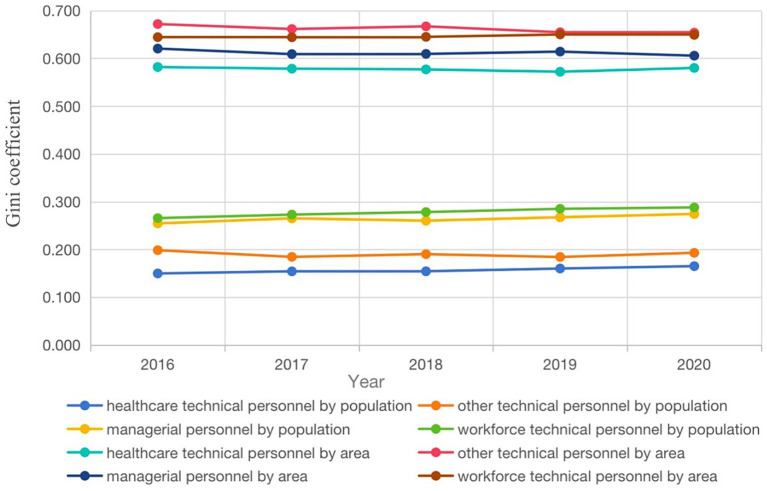
The G of the allocation of health human resources in CDCs in mainland China from 2016 to 2020.

Figure 3 illustrates the G for the allocation of health human resources within the China CDCs from 2016 to 2020, calculated based on population data. The G for health technical personnel, other technical personnel, managerial personnel, and workforce technical personnel ranged from 0.150 to 0.165, 0.199 to 0.193, 0.255 to 0.274, and 0.266 to 0.288, respectively.

The resource allocation for health technical personnel and other technical personnel appears to be relatively equitable, while the allocation for the other two categories, namely managerial personnel and workforce technical personnel, displays a tendency toward increasing equality relatively over the years.

According geographical data, the G for all four categories of personnel ranged from 0.582 to 0.580, 0.672 to 0.654, 0.620 to 0.606, and 0.644 to 0.649, respectively. These coefficients indicate that resource allocation for all four categories of personnel remained in an inequitable state, with G consistently higher than those calculated based on population data. With the exception of workforce technical personnel, the G of the other three categories of personnel decreased slightly year by year.

#### Theil index calculation results

Table 5 demonstrates that the T indices for the four personnel types, when weighted by geographical regions, are significantly higher than those weighted by population. Additionally, except for other technical personnel, the T for the other three personnel types, when weighted by population, exhibit an overall upward trend. When geographical regions are used as weights, the T indices for other technical personnel and managerial personnel, on the other hand, show an overall fluctuating downward trend. By decomposing the T, it can be observed that, under both weighting schemes, the contribution of intraregional disparities for managerial personnel and workforce technical personnel is higher than the contribution of interregional disparities for all years. When weighted by population, the variation in the contribution of intraregional disparities for these two personnel types ranges from 77.57 to 84.10% and from 72.64 to 77.57%, respectively. When geographical regions are used as weights, the variation in the contribution of intraregional disparities for these two personnel types ranges from 51.66 to 53.38% and from 51.90 to 55.38%, respectively. When geographical regions are used as weights, health technical personnel and other technical personnel exhibit interregional disparities with contributions higher than intraregional disparities.

**Table 5:** The *T* of the allocation of health human resources in China CDCs from 2016 to 2020.

Category	Configured by population					Configured by geographic area				
	2016	2017	2018	2019	2020	2016	2017	2018	2019	2020
**Healthcare technical personnel**										
*T*	0.0367	0.0388	0.0391	0.0415	0.0424	0.6093	0.6036	0.6023	0.5939	0.6103
*T_inter_*	0.0129	0.0149	0.0166	0.0180	0.0188	0.3324	0.3227	0.3149	0.3094	0.3138
*T_intra_*	0.0238	0.0239	0.0225	0.0234	0.0235	0.2768	0.2810	0.2874	0.2845	0.2965
**Other technical personnel**										
*T*	0.0648	0.0574	0.0616	0.0548	0.0589	0.8672	0.8326	0.8530	0.8035	0.8131
*T_inter_*	0.0083	0.0042	0.0053	0.0018	0.0019	0.5319	0.4933	0.4906	0.4765	0.4837
*T_intra_*	0.0565	0.0532	0.0564	0.0531	0.0570	0.3353	0.3393	0.3624	0.3270	0.3294
**Managerial personnel**										
*T*	0.1009	0.1093	0.1059	0.1110	0.1167	0.7133	0.6694	0.6692	0.6892	0.6580
*T_inter_*	0.0160	0.0245	0.0224	0.0217	0.0261	0.3433	0.3121	0.3235	0.3237	0.3162
*T_intra_*	0.0849	0.0848	0.0835	0.0893	0.0906	0.3700	0.3574	0.3457	0.3654	0.3418
**Workforce technical personnel**										
*T*	0.1217	0.1285	0.1331	0.1459	0.1432	0.7974	0.7969	0.8010	0.8260	0.8172
*T_inter_*	0.0273	0.0321	0.0323	0.0362	0.0392	0.3835	0.3777	0.3755	0.3686	0.3711
*T_intra_*	0.0944	0.0964	0.1008	0.1097	0.1040	0.4139	0.4191	0.4254	0.4575	0.4461

Table 6 shows that for the T of managerial personnel and workforce technical personnel calculated with population weights, the ranking of intraregional disparity contributions across the Eastern, Central, and Western regions is generally as follows: Central > Eastern > Western. As for healthcare technical personnel, the ranking of disparity contributions across regions is: Western > Eastern > Central. For other technical personnel, the ranking of disparity contributions across regions is: Central > Western > Eastern. When geographical regions are used as weights, the regions with the highest intraregional disparity contributions for healthcare technical personnel, other technical personnel, and managerial personnel are consistently in the Western region. For workforce technical personnel, the region with the highest intraregional disparity contribution is the Central region.

**Table 6:** The contribution rate of the T of the health human resources allocation of China CDCs from 2016 to 2020.

Category	Contribution rate by population/%					Contribution rate by geographic area/%				
	2016	2017	2018	2019	2020	2016	2017	2018	2019	2020
**Healthcare technical personnel**										
Eastern Region	17.19	17.28	14.75	11.20	15.98	7.41	7.77	8.36	8.93	8.84
Central Region	16.17	14.78	14.01	20.18	11.48	7.49	7.53	7.59	5.86	7.09
Western Region	31.45	29.51	28.77	25.12	28.12	30.54	31.24	31.77	33.10	32.65
**Other technical personnel**										
Eastern Region	18.73	12.59	11.45	16.26	22.40	9.73	9.48	9.11	8.00	9.34
Central Region	42.63	50.51	50.98	50.14	46.58	11.46	12.16	13.46	12.63	11.92
Western Region	25.91	29.54	29.04	30.35	27.78	17.47	19.10	19.92	20.06	19.25
**Managerial personnel**										
Eastern Region	34.56	28.18	28.51	27.81	28.57	9.23	6.54	6.35	7.67	6.45
Central Region	32.91	30.47	32.49	32.19	29.16	11.67	13.05	12.20	11.82	10.94
Western Region	16.63	18.92	17.90	20.43	19.93	30.97	33.79	33.11	33.53	34.55
**Workforce technical personnel**										
Eastern Region	13.21	12.40	13.31	10.76	13.18	2.97	2.54	2.88	2.98	2.03
Central Region	54.02	53.70	53.00	57.66	51.92	25.28	26.35	26.56	28.65	28.54
Western Region	10.33	8.91	9.41	6.74	7.53	23.66	23.71	23.67	23.75	24.02

## Discussion

Organizations similar to global disease prevention and control centers currently focus their research and efforts on strengthening health equity, addressing infectious diseases, and tackling challenges related to healthcare human resources, especially in the context of the ongoing pandemic and other health threats (32–34). The outbreak of the COVID-19 pandemic has underscored the importance of public health professionals equipped with advanced skills in data analysis and monitoring to track disease transmission and formulate effective control measures (35). Faced with new pathogens and other threats, adaptability and innovation are essential qualities for public health professionals to develop new methods to combat epidemics (36). The contributions made by Chinese public health authorities in the fight against the COVID-19 pandemic have been multifaceted, contributing to the global health and well-being, aligning with the vision and goals of sustainable development outlined by the WHO.

This study analyzed the basic characteristics of public health personnel and revealed that from 2016 to 2020, there was an initial decline followed by growth in the total number of personnel at the China CDCs, potentially influenced by the global COVID-19 pandemic. The pandemic significantly increased the workload of public health agencies worldwide, necessitating the recruitment of more personnel, especially in key fields such as epidemiology, public health, and infectious diseases.

The WHO reports that due to the COVID-19 pandemic, healthcare departments are facing significant human resource challenges. According to a report from 2023, at least 55 countries are experiencing severe shortages of medical personnel. This issue is particularly acute in Africa, where 37 countries are grappling with shortages, jeopardizing their ability to achieve universal health coverage by 2030, a crucial sustainable development goal (37).

In the post-pandemic era, it is recommended that CDCs in developed countries establish closer collaborations with developing nations, providing technical expertise, training, and financial support. For example, through international aid programs, they can offer specialized training and educational resources. By partnering with international health organizations like the WHO, they can help establish regional training centers in economically disadvantaged countries to enhance the skills and knowledge of local healthcare professionals, promoting the sharing of human resources.

From 2016 to 2020, the educational level of personnel at the China CDC has significantly improved, with an increasing proportion of staff holding undergraduate and graduate degrees, and a decreasing proportion of staff having only high school education or below. Specifically, the proportion of staff with undergraduate and graduate degrees increased to 1.25 times that of 2016, and the proportion of healthcare technical personnel with senior professional titles also rose. Overall, there has been an enhancement in human resource quality, primarily reflected in the increase in educational attainment. The reason for this trend is that after the outbreak of COVID-19, governments at all levels have paid unprecedented attention to the construction of disease control teams. China CDCs have also recruited a large number of personnel with a bachelor’s degree or above. At the same time, relying on the resources of colleges and universities, CDC at all levels is encouraged to establish cooperative relationships with colleges and universities with preventive medicine and public health related majors, and pilot “order-type” orientation training is carried out to provide talent reserve to meet the needs of disease control work. The improvement of the educational level of the personnel of the China CDC is not only a passive reflection of the improvement of the overall education level of the society, but also a positive reflection of the construction of “healthy China.” Additionally, the proportion of staff with over 30 years of work experience has increased. This suggests that the talent pool at the China CDCs is gradually seeing an influx of highly qualified personnel while experiencing a decrease in those with lower qualifications. However, from a more comprehensive perspective, it has not yet completely altered the current situation of a relatively low educational level and a low proportion of senior professional title holders among public health personnel in China. Additionally, there is an ongoing challenge related to the aging of personnel in the field. It is recommended to establish a standardized and effective training mechanism to continuously enhance the professional competence and overall qualities of CDC personnel, promoting the high-quality development of regional disease control agency staff. The government should formulate specific strategies for human resource development to ensure the sustained and effective operation of the public health system.

Through an analysis of the *HRDI*, it is evident that there are disparities in the allocation of human resources across regions with different levels of economic development. In 2020, the *HRDI* for healthcare technical personnel and other technical personnel in Shanghai was approximately 10.78 times and 36.10 times that of the Tibet, respectively. For managerial personnel in Tianjin, the *HRDI* was 17.00 times that of the Tibet, and for workforce technical personnel in Henan, it was 21.69 times that of the Tibet.

It is observed that the Eastern regions consistently maintain higher levels of human resource density in public health, while the Western regions exhibit lower levels. This discrepancy may be attributed to variations in regional economic development levels and population density. The eastern region is relatively economically developed, able to provide greater support for talent incentives, and has a higher demand for healthcare. Moreover, economically prosperous areas are more attractive to healthcare professionals, offering more development opportunities. On the other hand, regions with lower economic development levels struggle to attract and retain healthcare professionals due to limited opportunities. The Chinese government document “Guiding Opinions on Staffing Standards for Disease Control and Prevention Centers” suggests that “Provinces and autonomous regions with an area of more than 500,000 square kilometers and a population density of less than 25 people per square kilometer may be determined on the basis of a ratio not higher than 3 ‰ of the permanent resident population of the region” (38). For example, Qinghai Province should have around 1,800 CDC staff based on this guideline, but in 2019, the approved number was only about 1,599. In response to this situation, local governments have paid attention to gradually increasing the proportion of disease control professionals at all levels, rationally increasing the proportion and number of posts with senior professional titles, and improving the training, access, assessment and evaluation mechanisms for disease control practitioners. To address these disparities, future government healthcare investments should focus on balancing regional disparities and appropriately tilting toward areas with lower economic development levels. Meanwhile, in regions with lower economic levels, motivation and retention of local healthcare professionals can be encouraged by offering competitive salaries, career development opportunities, and working conditions. It is recommended that the optimization of the regional human resource allocation of within China CDCs take into account factors such as regional population size, healthcare needs, and the workload of disease control agencies, to promote fair and equitable distribution of personnel across regions.

The results of the decomposition of T show that the inequality in the distribution among the four types of people is predominantly caused by intra-regional disparities, particularly when weighted by population. Utilizing geographical weights further elucidates that the unequal distribution of managerial personnel and workforce technical personnel is mainly caused by intra-regional disparities. On the whole, the main reason for the unfair distribution of human resources is the intra-regional difference between the central and western regions. In addition, when calculated according to geographical weights, the contribution rate of intra-regional differences in the central and western regions is particularly significant. This is likely because central and western regions like Tibet and Xinjiang are subject to governmental allocation of human resources primarily based on population metrics. This occurs despite their distinct characteristics of sparse populations and vast areas. Such approach overlooked the significant geographical challenges unique to these areas, inadvertently amplifying the development difference between them and the more densely populated eastern regions. Another reason might be that government health departments typically use the quantity of healthcare resources per 1,000 people as a standard for regional planning and allocation, with insufficient attention to the geographical availability of healthcare resources (39). This situation is not unique to China, the uneven distribution of health human resources is a global problem, and countries have taken corresponding measures to solve this problem. Thailand introduced a “mandatory public service policy,” requiring medical graduates to work in public healthcare institutions for 3 years after graduation. Additionally, Thailand reformed medical education to recruit, train, and employ healthcare workers in rural areas, increased government funding for grassroots medical institutions, and raised salaries for healthcare workers. Australia established a classification standard for remote areas, breaking administrative boundaries and addressing uneven distribution through targeted training and temporary replacement systems for rural doctors. Cuba implemented a rotational service system, requiring medical graduates to serve in remote rural areas for 2 years as an exchange for free higher education. India relies on both government and non-governmental organizations, establishing mobile medical teams in various districts to improve the health conditions of the rural poor (40). Unequal geographic distribution of healthcare resources can limit the equitable allocation and management of these resources, ultimately affecting the fairness and accessibility of healthcare services. Therefore, it is imperative for the formulation of new-era CDC human resource planning to incorporate considerations of regional population size, healthcare needs, and the workload of disease control agencies, aiming to promote fair and rational distribution of personnel across regions.

Furthermore, other countries can also draw inspiration from China’s model, such as setting overall goals for human resource development in CDCs, updating regional human resource data on a regular basis, making timely adjustments to policies and resource allocation strategies, and conducting regular assessments.

## Conclusion

In recent years, efforts by these agencies and China CDCs have achieved significant success. However, there are still differences in the distribution of CDCs human resources in different economic development regions in China, with fairness in allocation based on population being prioritized over fairness based on geographical regions. In the future, the Chinese government should pay particular attention to the impact of intraregional disparities on the allocation of human resources within CDCs and continuously work to improve the geographic accessibility of CDC services. Although this study provides comprehensive analysis and insights, there are several limitations. First, the study examined the differences in human resource allocation between regions, but did not consider the differences within provinces, which may have significant differences within some provinces. The use of Theil index to analyze intra-regional differences may mask regional cooperation and resource spillover effects. Considering the vast territory of China and the high cost of inter-provincial flow, the resources of developed provinces mainly attract neighboring regions, so the resource spillover effect is relatively small and will not significantly affect the conclusion. The study covers the period 2016 to 2020 and does not reflect the impact of post-2020 policy changes, economic conditions or public health emergencies such as COVID-19. Future studies should consider more recent data to provide up-to-date insights. Furthermore, HRDI has its limitations as a composite indicator and may not fully capture the quality of human resources, such as the specific skills and competencies of healthcare personnel, which are critical to the public health response. This study mainly focuses on the impact of economic differences on resource allocation, but does not delve into non-economic factors such as policy implementation efficiency. The practical challenges in implementing the proposed policy recommendations, which could significantly affect the effectiveness of the solutions, are not discussed. By acknowledging these limitations, this study highlights the need for continuous data collection, comprehensive analysis, and adaptive policy development to ensure equitable distribution of human resources for health in different regions of China. Future studies should integrate more data sources, expand the time frame, and consider a wider range of influencing factors.

## Data Availability

Publicly available datasets were analyzed in this study. These datasets can be found at: http://www.nhc.gov.cn/wjw/index.shtml and https://www.stats.gov.cn/.

## References

[1] Na L Lieyu H Hao C Jia Z Yuan W Yan G. The current status of information system construction in the German disease control system and its implications for China [J/OL]. Chin J Prev Med. (2024) 1:123–128. doi: 10.16506/j.1009-6639.2024.01.022

[2] Zhaoyan T Yi X Zuyang L Li L Mengting M. Considerations on the construction of nutrition and food hygiene disciplines in disease prevention and control centers in the context of healthy China. Chin J Public Health Manage. (2023) 39:799–801. doi: 10.19568/j.cnki.23-1318.2023.06.0011

[3] Zhao H. Evolutionary game analysis of collaborative prevention and control for public health emergencies. Sustain For. (2022) 14:89. doi: 10.3390/su142215089

[4] Yun J Yuhong Z Hongbao H Huafeng Y. Comprehensive evaluation of research capabilities of disease prevention and control institutions based on entropy weighted TOPSIS method. Chin J Public Health Manage. (2023) 39:646–8. doi: 10.19568/j.cnki.23-1318.2023.05.0012

[5] Yipeng L Fan C Xiaoqiong Z Haidong K Hongjie Y Chaoxin W. Current status and prospects of primary public health Services in China in the new era. Chin Gen Pract. (2022) 20:1631–4. doi: 10.16766/j.cnki.issn.1674-4152.002666

[6] Shao S Zhou Z Li Y Liu S Lu L Hou S. Experiences and practices in the current prevention and control of the novel coronavirus pneumonia in China. Disaster Med Public Health Prep. (2021) 15:e7–e16. doi: 10.1017/dmp.2020.173, PMID: 32466822 PMC7484311

[7] Na T Ran C Guangfu J Jianming W Yankai X Zhibin H. Current status of public health academic disciplines in Chinese universities participating in scientific research on novel coronavirus pneumonia. Chin J Dis Control. (2020) 24:621–7. doi: 10.16462/j.cnki.zhjbkz.2020.06.001

[8] Davis XM Rouse EN Stampley C. Preparing the CDC Public Health Workforce for Emergency Response. J Homeland Secur Emerg Manage. (2020) 18:1–21. doi: 10.1515/jhsem-2019-0021

[9] Brencic DJ Pinto M Gill A Kinzer MH Hernandez L Pasi OG. CDC support for global public health emergency management. Emerg Infect Dis. (2017) 23:S183–9. doi: 10.3201/eid2313.170542, PMID: 29155652 PMC5711305

[10] Yi L Li L Yucun N Chunhong S. Several considerations on the establishment of a new public health and preventive medicine system in national level. Zhonghua Yu Fang Yi Xue Za Zhi. (2020) 54:469–474. Chinese. doi: 10.3760/cma.j.cn112150-20200221-00159 32388945

[11] Liburd LC Hall JE Mpofu JJ Williams SM Bouye K Penman-Aguilar A. Addressing health equity in public health practice: frameworks, promising strategies, and measurement considerations. Annu Rev Public Health. (2020) 41:417–32. doi: 10.1146/annurev-publhealth-040119-094119 31900101

[12] Li H Gu S Gong H Zhang R, International comparison of health human resource allocation. In: *2020 Chinese control and decision conference (CCDC)*.(2020).

[13] Wu F Chen W Lin L Ren X Qu Y. The balanced allocation of medical and health resources in urban areas of China from the perspective of sustainable development: a case study of Nanjing. Sustain For. (2022) 14:6707. doi: 10.3390/su14116707

[14] Guichang Z. Achieving the United Nations millennium development goals: China's contribution and experience. Contempor World Soc. (2015) 4:144–8. doi: 10.16502/j.cnki.11-3404/d.2015.04.023

[15] Li D Luo M. The Chinese experience and cooperation plan for building a global community of health for all. Journal of Wuhan University of Science and Technology: Social Science Edition. (2021) 23:8. doi: 10.3969/j.issn.1009-3699.2021.01.003

[16] Ministry of Health of the People's Republic of China. China health statistics yearbook. Beijing: Peking Union Medical College Press (2012). 2012 p.

[17] Dai G Li R Ma S. Research on the equity of health resource allocation in TCM hospitals in China based on the Gini coefficient and agglomeration degree: 2009-2018. Int J Equity Health. (2022) 21:7. doi: 10.1186/s12939-022-01749-7, PMID: 36199086 PMC9534739

[18] Wang Y Gong X. Analyzing the difference evolution of provincial energy consumption in China using the functional data analysis method. Energy Econ. (2022) 105:105753. doi: 10.1016/j.eneco.2021.105753

[19] Sun B Wang J Zeng X Zhang H Zhao H Li X. Study on the equity of health resource allocation in Heilongjiang reclamation area. China Health Rev. (2023) 40:357–360.

[20] Zheng X Ling F. Application of HRDI in health resource evaluation in Sichuan ethnic areas. China Health Manag. (1996) 12:665–7.

[21] Lin J Gong J Shen S. Equity of healthcare resource allocation in Guangdong Province during the “Twelfth Five-Year Plan” period. Prev. Med. (2018) 45:1048–1051.

[22] Mao Y Zhu B Liu J Jing P Wu J Li Y. Analysis of the equity in health human resource allocation in Western China: Based on the assumption of resource homogeneity. Chin. Health Econ. (2015) 34:31–34.

[23] Wei Y He J. Study on the equity of health resource allocation in the process of China’s medical reform: An analysis based on the reasonable accountability framework. Morality and Civilization. (2012) 6:120–125. doi: 10.13904/j.cnki.1007-1539.2012.06.001

[24] Yunfeng X Hongpei Z Xiue D Wei Z. Research on the fairness of primary healthcare resource allocation in China in the past decade. Health Econ Res. (2023) 40:1–6. doi: 10.14055/j.cnki.33-1056/f.2023.06.005

[25] Maihong H Lin W Jia H Yi W. A evaluation study on the equity of health resources of population distribution based on Lorenz curve and Gini coefficient. Northwest Popul J. (2013) 34:27–31. doi: 10.15884/j.cnki.issn.1007-0672.2013.02.002

[26] Xu Z Lei X. Analysis on equity of health human resource allocation in Hubei Province based on the Gini coefficient and a HRDI model. J Phys. (2021) 1941:012063. doi: 10.1088/1742-6596/1941/1/012063

[27] Shang S Shang S. Estimating Gini coefficient from grouped data based on shape-preserving cubic Hermite interpolation of Lorenz curve. Mathematics. (2021) 9:551. doi: 10.3390/math9202551

[28] Yu Z Li X Mu F Meng Q Wu T He L. Comparison of different calculation formulas for the Theil index. Chinese Journal of Health Statistics. (2020) 37:124–126.

[29] Kranzinger S. The decomposition of income inequality in the EU-28. Empirica. (2020) 47:643–68. doi: 10.1007/s10663-019-09450-9

[30] Ma Y Xiao P Yu L Ni H Huang S Wang M. The allocation and fairness of health human resources in Chinese maternal and child health care institutions: a nationwide longitudinal study. BMC Health Serv Res. (2023) 23:5. doi: 10.1186/s12913-023-09076-5 36782193 PMC9926631

[31] Yu H Yu S He D Lu Y. Equity analysis of Chinese physician allocation based on Gini coefficient and Theil index. BMC Health Serv Res. (2021) 21:6348. doi: 10.1186/s12913-021-06348-w PMC8115393 33980223

[32] Centers for Disease Control and Prevention. CDC Global Health Strategy. (2024a). Available online at: https://archive.cdc.gov/#/details?url=https://www.cdc.gov/globalhealth/strategy/default.htm

[33] Centers for Disease Control and Prevention. CDC Global Health Equity. (2024b). Available online at: https://www.cdc.gov/global-health-equity/php/?CDC_AAref_Val=https://www.cdc.gov/globalhealth/equity/home.html (Accessed February 10, 2024).

[34] Samuel L. New CDC report: Striving Toward Global Health Equity. CDC Foundation. (2023). Available online at: https://www.cdcfoundation.org/blog/new-cdc-report-striving-toward-global-health-equity (Accessed February 10, 2024).

[35] Mheidly N Fares NY Fares MY Fares J. Emerging health disparities during the COVID-19 pandemic. Avicenna J Med. (2024) 13:60–4. doi: 10.1055/s-0042-1759842 PMC10038746 36969348

[36] Daniel B Jingwei HA Ramesh M. COVID-19, crisis responses, and public policies: from the persistence of inequalities to the importance of policy design. Polic Soc. (2023) 2:21. doi: 10.1093/polsoc/puac021

[37] United Nations News. WHO: 55 countries face healthcare workforce losses due to COVID-19. (2023). Available online at: https://news.un.org/zh/story/2023/03/1116147 (Accessed February 10, 2024).

[38] Chinese Center for Disease Control and Prevention. Guiding Opinions on Staffing Standards for Disease Control and Prevention Centers. (2024). Available online at: https://m.chinacdc.cn/xwzx/zxyw/ (Accessed February 10, 2024).

[39] Dong E Sun X Zhang L Xu J Xu T Wang T. Differences in regional distribution and inequality in health-resource allocation on institutions, beds, and workforce: a longitudinal study in Shanghai, China. Arch Public Health. (2020) 79:78. doi: 10.1186/s13690-021-00597-1, PMID: 34001268 PMC8130126

[40] Peng L Huiying F Hongkun M Mingxin B Mingli J. Discussion on the development of healthcare human resources in China. Soft Sci Health. (2017) 31:4. doi: 10.3969/j.issn.1003-2800.2017.04.001

